# Novel rapid method for identifying and quantifying pathogenic bacteria within four hours of blood collection

**DOI:** 10.1038/s41598-023-50864-0

**Published:** 2024-01-12

**Authors:** Akio Miyakoshi, Hideki Niimi, Tomohiro Ueno, Masahiro Wakasugi, Yoshitsugu Higashi, Yuki Miyajima, Masashi Mori, Homare Tabata, Hiroshi Minami, Akinori Takaoka, Atsushi Hayashi, Yoshihiro Yamamoto, Isao Kitajima

**Affiliations:** 1 Department of Ophthalmology, Toyama University Hospital, Toyama, Japan; 2 Clinical Laboratory Center, Toyama University Hospital, 2630 Sugitani, Toyama, 930-0194 Japan; 3 Disaster and Emergency Center, Toyama University Hospital, Toyama, Japan; 4 Department of Clinical Infectious Diseases, Toyama University Hospital, Toyama, Japan; 5 Research Institute for Bioresources and Biotechnology, Ishikawa Prefectural University, Nonoichi, Japan; 6 Life Science Center, Hokkaido Mitsui Chemicals, Inc., Sunagawa, Japan; 7 Institute for Genetic Medicine, Hokkaido University, Sapporo, Japan; 8 Administrative Office, University of Toyama, Toyama, Japan

**Keywords:** PCR-based techniques, Diagnostic markers, Bacterial infection, Laboratory techniques and procedures

## Abstract

Sepsis is life-threatening organ dysfunction and is considered a major cause of health loss. However, since the current biomarkers of sepsis reflect the host’s immune response to microorganisms, they would inevitably cause a time-lag. This means that there is still no truly reliable biomarker of sepsis. In the present study, we developed a novel method for identifying and quantifying unknown pathogenic bacteria within four hours of sample collection. The most important point of this study is that the novel method can be used to determine the number of bacteria in a sample as a novel biomarker of infectious diseases. Indeed, based on the number of bacteria, we were able to accurately estimate the severity of microbial infection. Furthermore, using the time-dependent changes in the number of bacteria, we were able to monitor the therapeutic effect accurately. The rapid identification and quantification of bacteria may change our approach to medical care.

## Introduction

Sepsis refers to the life-threatening organ dysfunction induced by a dysregulated host response to infection^1^ and is considered a major cause of health loss. In 2017, an estimated 48.9 million incident cases of sepsis were recorded worldwide, and 11.0 million sepsis-related deaths were reported, representing 19.7% of all global deaths^2^. In patients suspected of having sepsis, initial appropriate antibiotic treatment results in a favorable outcome and a significant decrease in the mortality rate^3,4^. However, in order to select the optimum initial antimicrobial treatment, the early identification of pathogenic microorganisms is critical. To address this problem, we previously reported the development of a novel rapid, easy, cost-effective method of identifying a broad range of pathogenic bacteria (> 100 bacterial species) using a real-time polymerase chain reaction (PCR)-based system; we named this novel method the Tm mapping method^5–7^.

In the treatment of sepsis, it is difficult to judge the best timing to stop antibiotic treatment or even whether a given antibiotic treatment is actually appropriate. To make accurate judgments, we need biomarkers that strictly reflect the severity of sepsis. The biomarkers currently used to reflect the severity of sepsis include procalcitonin, presepsin, and C-reactive protein (CRP) levels; the body temperature (BT); and the white blood cell (WBC) count^8^. However, while these biomarkers are correlated with the severity of sepsis to some extent, they do not always accurately reflect the severity at a particular point in time. For example, procalcitonin cannot reliably differentiate sepsis from other non-infectious causes of systemic inflammatory response syndrome in critically ill adult patients^9^, and presepsin concentrations increase with age and kidney dysfunction, so their interpretation might be altered in elderly patients or those with an impaired renal function^10^. Thus, there is still no truly reliable biomarker of sepsis.

The fluctuations in the biomarkers mentioned above are due to host defenses against microbial infection. In the present study, as a novel biomarker to accurately judge the severity of sepsis, we suggest “the number of bacteria in a blood sample”, which is not a result of the host defense but direct information about the pathogen itself. The quantitative detection of a specific type of bacteria in a clinical sample (e.g. *Borrelia burgdorferi*^11^, *Porphyromonas gingivalis*^12^, *Mycobacterium tuberculosis*^13^, *Streptococcus pneumoniae*^14^, *Mycoplasma ovipneumoniae*^15^, *Staphylococcus aureus*^16^, *Enterococcus faecalis*^16^, and *pneumococcal pneumonia*^17^, etc.) has been reported previously. However, the quantitative detection of unknown bacteria in a clinical sample is quite difficult technically. In fact, there have been no reports of methods to accurately quantify unknown pathogenic bacteria in a clinical sample.

If the bacterial species are unknown, a broad range of bacteria can be detected using bacterial universal primers targeting highly conserved regions in the bacterial 16S ribosomal RNA (rRNA) gene^18^. The main problem associated with bacterial universal PCR is the achievement of the sensitive and reliable detection of bacteria without any bacterial DNA contamination. This is because the reagents used in the PCR process, especially stock solution of the thermostable DNA polymerase^19–22^, are usually contaminated with trace amounts of bacterial DNA. Whenever pathogenic bacteria are present in very small quantities, bacterial DNA contamination becomes a major problem. Since even low-copy-number contamination will be amplified with bacterial universal PCR, there are bound to be issues with obtaining low detection limits in a clinical sample.

To address this, we developed a eukaryote-made thermostable DNA polymerase that is free from bacterial DNA contamination^23^. Using this thermostable DNA polymerase, the sensitive and reliable detection of bacteria is feasible, making the quantitative detection of a small amount of bacteria in a clinical sample possible.

One more problem in the quantitative detection of unknown bacteria is the variation of the 16S rRNA operon copy number in genomes among bacterial species. Quantification results using bacterial universal primer do not reflect actual bacterial concentrations unless adjusted according to the pathogen’s 16S rRNA operon copy number. Our quantification method is based on the melting temperature (Tm) mapping method for rapidly identifying the dominant bacteria in a clinical sample^5^. In the novel quantification method, quantification results can be adjusted according to the 16S rRNA operon copy number of the pathogen identified by the Tm mapping method.

We herein report the development of a novel rapid identification and quantification method of unknown bacteria in a clinical sample using a real-time PCR system. We also report the importance of the number of bacteria in a septic blood sample compared to other current biomarkers.

## Results

### The rapid identification and quantification method workflow

**Figure 1:**
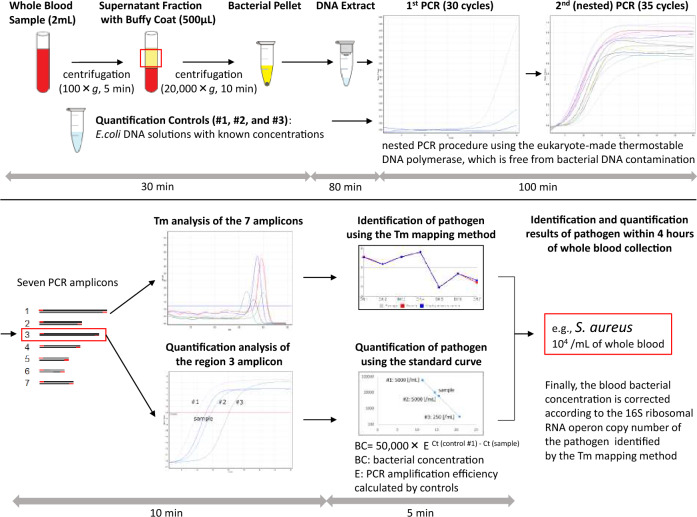
Workflow of the novel rapid method for identifying and quantifying unknown pathogenic bacteria in sepsis within four hours of whole-blood collection. *This figure conceptually shows the workflow of the novel method, and the individual values in the figure have no meaning.

The workflow of the rapid identification and quantification method for unknown pathogenic bacteria developed in our laboratory is shown in Fig. 1. This method consists of five major steps.

First, bacterial DNA is extracted directly from a clinical sample (2 mL of a whole blood sample, etc.) as a template for PCR. Three quantification standards (*Escherichia coli* DNA solutions) with different known concentrations (the number of bacteria/mL) measured by flow cytometry are also prepared in advance. Step two involves nested PCR using the seven bacterial universal primer sets; these primers can detect almost all species of bacteria. In order to achieve accuracy in this PCR step, we developed a eukaryote-made thermostable DNA polymerase which is free from bacterial DNA contamination^23^. This eukaryote-made thermostable DNA polymerase is a recombinant polymerase manufactured using eukaryotic (yeast) host cells. Employing this DNA polymerase in bacterial universal PCR, sensitive and reliable detection of bacteria without false-positive results is feasible, thereby making it possible for PCR to identify and quantify bacterial isolates directly from patient samples. After the nested PCR procedure is performed, seven (or fewer) PCR amplicons are obtained. In step three, the seven Tm values are acquired by analyzing the amplicons. Step four involves mapping the seven Tm values in two dimensions. The plot creates a unique species-specific shape, known as the Tm mapping shape. By comparing the Tm mapping shape to the shapes in the database, the bacterial isolates can be rapidly identified. We named this novel method the “Tm mapping method” and described it in detail previously^5^. In step five, the bacterial concentration is measured in conversion as *E. coli* using the standard curve formed by Ct values (region 3 amplicon) of the three different quantification standards (*E. coli* DNA) with known concentrations. Finally, the bacterial concentration is corrected according to the 16S ribosomal RNA operon copy number (Supplemental Table S1) of the bacteria rapidly identified by the Tm mapping method. As a result, identification and quantification results of pathogenic bacteria in a clinical sample can be obtained within four hours of sample collection.

### Establishment of the quantification method

**Figure 2:**
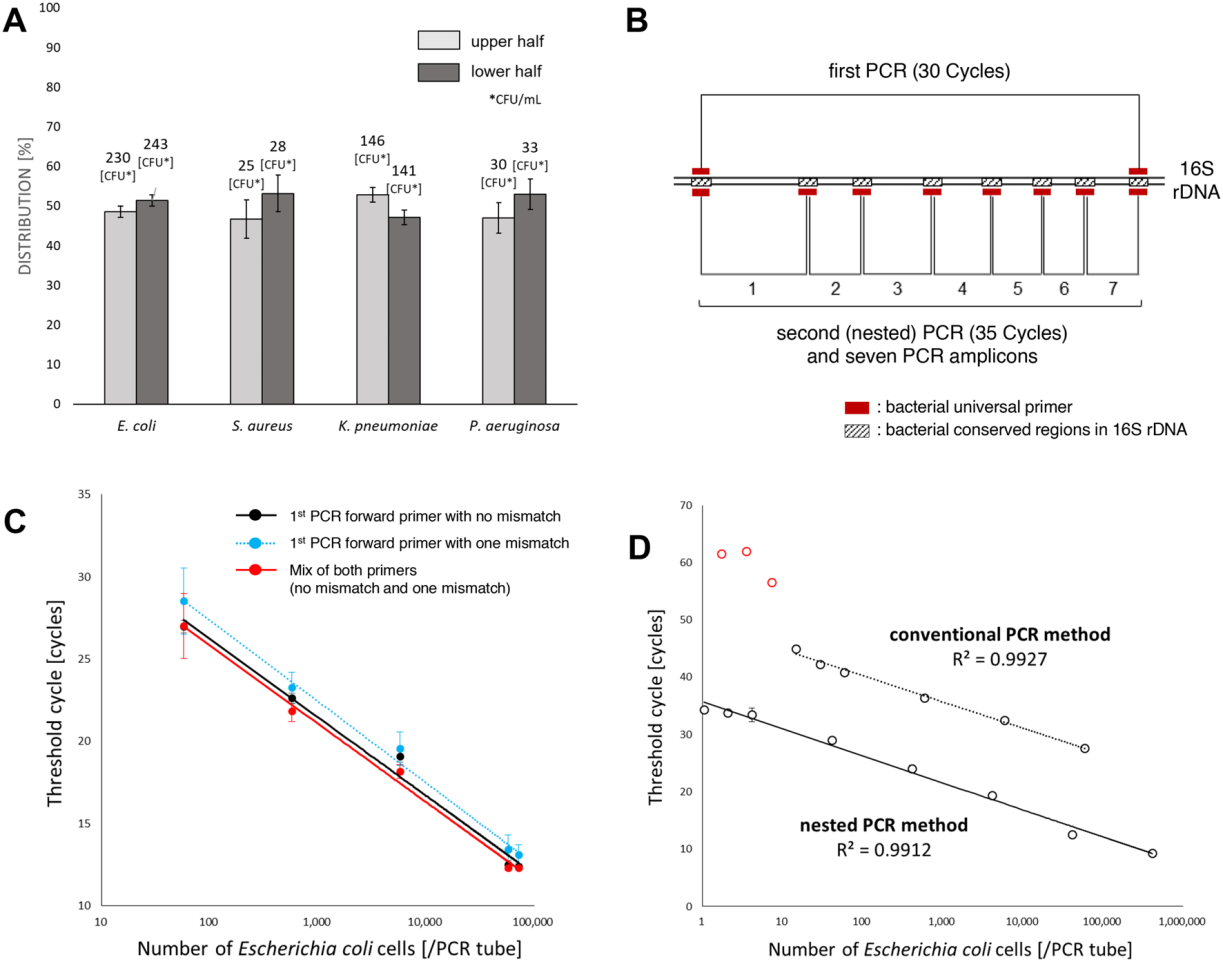
Establishment of the quantification method. (**A**) Distribution of bacteria (CFU/mL) in saline after low-speed centrifugation. The bacterial concentration in the upper half and the lower half were examined after low-speed centrifugation (100×*g*, 5 min). A total of 2 mL of bacterial suspension (*E. coli*, *S. aureus*, *K. pneumoniae* and *P. aeruginosa*) in saline was centrifuged, and the colony-forming unit (CFU) value was measured in the upper and lower half (1 mL each). The figure shows the ratio (%) of each half. Error bars indicate triplicate testing. (**B**) The primer designs. Nested PCR is performed using seven bacterial universal primer sets, and then the seven PCR amplicons (Tm values) are obtained. The pathogenic bacteria are identified using the seven Tm values of region 1 to 7 amplicons, and the bacterial concentration is measured using the region 3 amplicon alone. (**C**) Standard curves generated using three different primers. We generated standard curves by conducting an analysis of serial dilutions of *E. coli* DNA using primers as follows: 1st PCR forward primer with no mismatch against *E. coli*, 1st PCR forward primer with one mismatch against *E. coli*, and a mix of both 1st PCR forward primers (no mismatch : one mismatch = 1: 1). The 1st PCR reverse primer was the same in all cases. These results indicated that the quantification result using the 1st PCR forward primer with 1 mismatch was around 25% lower than that using the 1st PCR forward primer with no mismatch. In contrast, the quantification result using the mixed 1st PCR forward primers was almost the same as that using the 1st PCR forward primer with no mismatch. Error bars indicate triplicate testing. (**D**) The comparison of the regression lines generated by serial dilution of a known amount of *E. coli* DNA using conventional PCR method and nested PCR method. The regression lines calculated for the datum points are shown. The linear correlation between the Ct values and the logarithm of the number of *E. coli*/PCR tube (R^2^ value) was > 0.99. Error bars indicate triplicate testing.

The first step in developing the quantification method is to reduce the loss of bacterial cells as much as possible during the isolation of bacteria from whole blood samples. To isolate bacteria from red blood cells, the blood sample is slightly centrifuged (100×*g*, 5 min) to spin down the red blood cells only, and the resulting supernatant fraction with buffy coat (500 μL) is used. In this step, the distribution of bacteria (*E. coli*, *S. aureus*, *K. pneumoniae* and *P. aeruginosa*) in plasma is almost unchanged after low-speed centrifugation (Fig. 2A). After pelletization of the supernatant fraction with buffy coat, to maintain constant (maximize) DNA extraction efficiency regardless of bacterial species, we use not only Proteinase K but also small beads that assist in lysing bacterial cell walls thoroughly (see the Method section).

The second step involves the performance of accurate quantitative real-time PCR of bacteria in a blood sample. The primer designs are shown in Fig. 2B, and using region 3 amplicon, the quantification analysis is performed. In this step, it is necessary to eliminate the adverse effect of the base sequence differences between the primers and bacterial target regions, which affect the Ct values. As a countermeasure for this problem, we use mixed 1st PCR forward primers (Fig. 2C). Regarding the 16S bacterial conserved region targeted by the 1st PCR forward primer, there are mainly two kinds of base sequences among bacterial species (AGAGTTTGATCATGGCTCAG or AGAGTTTGATCCTGGCTCAG). We generated standard curves by an analysis of serial dilution of *E. coli* DNA using the primers as follows: 1st PCR forward primer with no mismatch against *E. coli* (AGAGTTTGATCATGGCTCAG), 1st PCR forward primer with one mismatch against *E. coli* (AGAGTTTGATCCTGGCTCAG), a mix of both 1st PCR forward primers (no mismatch : one mismatch = 1 : 1). The 1st PCR reverse primer is the same in all cases. The quantification result using the 1st PCR forward primer with one mismatch was around 25% (PCR amplification efficiency/cycle = 62%, Ct difference = 1.2) lower than when using the 1st PCR forward primer with no mismatch. In contrast, the quantification result using the mixed 1st PCR forward primers was almost the same as when using the 1st PCR forward primer with no mismatch. Moreover, in the 2nd PCR, we purposely use the region 3 amplicon to quantify bacteria. This is because the base sequences of the region 3 primers are the almost completely conserved among all bacterial species and does not affect the Ct values. As a result, we concluded that, when using the mixed 1st PCR forward primers and the region 3 primers, accurate quantitative real-time PCR of bacteria is feasible, regardless of bacterial species.

In the third step, for sensitive and accurate quantification of a small number of bacteria, we adopted the nested PCR method. In addition, to eliminate adverse effects on quantification due to primer-dimer formation, we set the temperature for fluorescence acquisition to 82 °C instead of the conventional 72 °C. With this ingenuity, the hydrogen bonds of the primer dimers are dissociated and broken, allowing the accurate quantification of only the target bacterial amplicons. To confirm the quantitative accuracy of the nested PCR method compared with conventional one-time quantitative PCR, we evaluated the regression lines generated by serial dilution of a known amount of *E. coli* DNA (Fig. 2D). As a result, the linear correlation between the Ct values and the logarithm of *E. coli* count/PCR tube (R^2^ value) was > 0.99. The nested PCR method was found to be more accurate than the conventional one-time quantitative PCR method for quantifying a small number of bacteria (1.0 to 10.0 *E. coli*/PCR tube). The limit of quantification for the nested PCR method was 1.0 genomic DNA of *E. coli*/PCR tube, and a wider linear dynamic range (1.0 to 4.1 × 10^5^ genomic DNA of *E. coli*/PCR tube) was established. Using this reliable range, the blood bacterial concentrations in conversion as *E. coli* are measured and finally corrected according to the 16S ribosomal RNA operon copy number of the pathogen. The measurement error of this quantification method is around ± 5% (Supplemental Table S2).

### Identification and quantification of pathogenic bacteria in septic blood samples

To evaluate the utility of the rapid method for identifying and quantifying pathogenic bacteria, we first tried to identify and quantify bacteria in blood samples of five healthy controls (Table 1) using the results of conventional blood culture, body temperature (BT, °C), WBC, CRP, presepsin, blood urea nitrogen (BUN), and creatinine (Cr). As a result, no bacteria were detected in the blood samples of any healthy controls (5 out of 5 controls), and those blood cultures showed no bacterial growth. In this case, “0 bacteria/mL of blood” means “ < 20 bacteria (calculated as *E. coli*)/mL of blood” based on the limit of detection (LOD) of this method.

**Table 1:** The identification and quantification results of bacteria in blood samples of five healthy controls.

Healthy controls	Identification results		Quantification results (mL of blood)	Laboratory data					
	Conventional culture method	Tm mapping method		BT (℃)	WBC (µ/L)	CRP (mg/L)	BUN (mg/dL)	Cr (mg/dL)	Presepsin (ng/mL)
1	No culture growth	None detected	0*	36.2	8540	1.0	14	0.90	159
2	No culture growth	None detected	0*	36.9	6810	7.5	16	0.78	206
3	No culture growth	None detected	0*	36.3	5370	0.1	21	0.62	130
4	No culture growth	None detected	0*	36.5	6760	0.8	13	0.72	119
5	No culture growth	None detected	0*	36.1	7430	0.2	12	1.05	189

*BT* body temperature,* WBC* white blood cells,* CRP* C-reactive protein,* BUN* blood urea nitrogen,* Cr* creatinine.

*A quantification result of “0 bacteria/mL of blood” means “ < 20 bacteria (calculated as *E. coli*)/mL of blood” based on the limit of detection (LOD) of this method.

**Figure 3:**
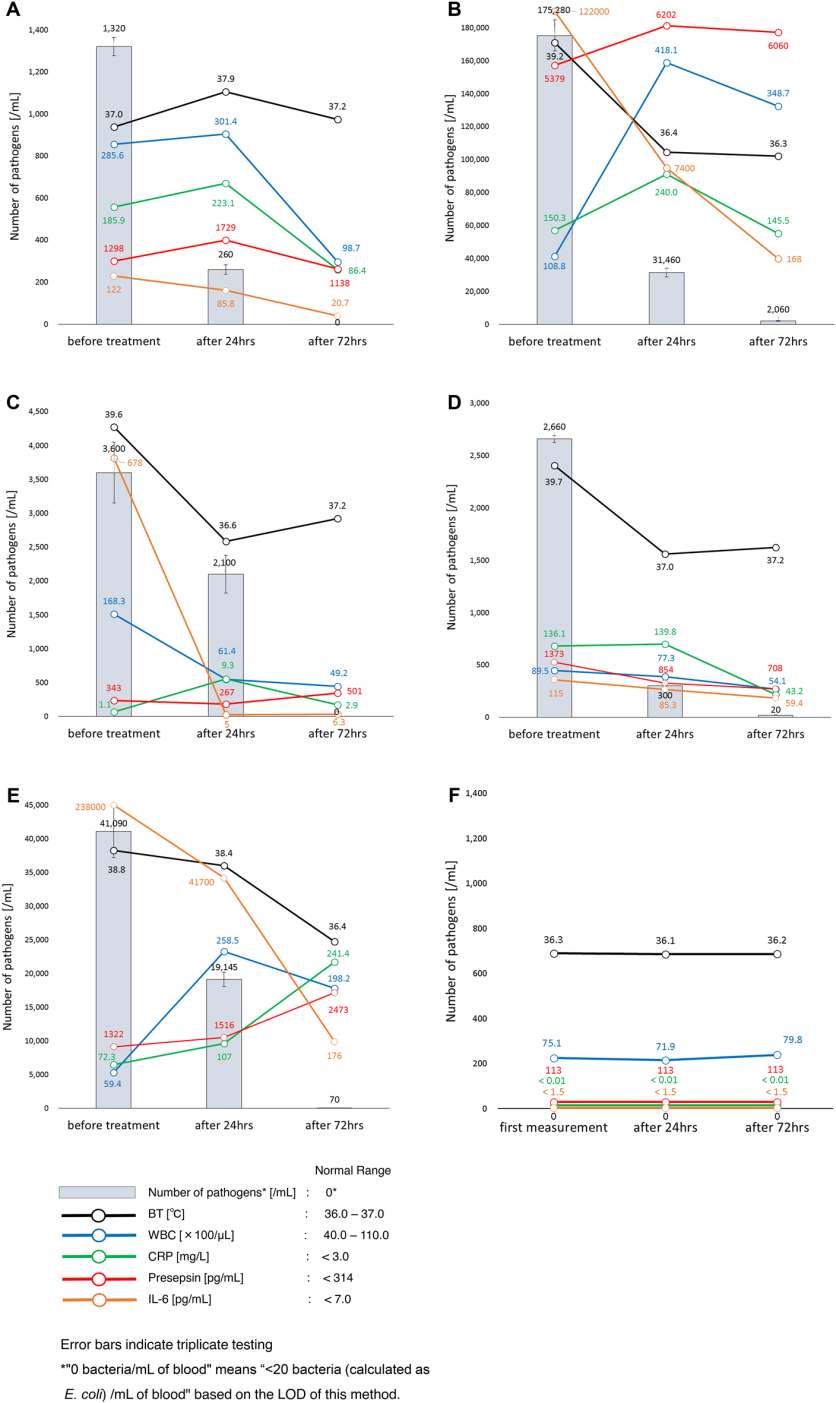
Time-dependent changes of the number of bacteria, BT, WBC, CRP, Presepsin and IL-6 in sepsis patients before antibiotic treatment and 24 and 72 h after. (**A**) Case 1: A 76-year-old woman was diagnosed with sepsis associated with urinary tract infection. The Tm mapping result (Difference Value = 0.29) was *E. coli*, and 2 days later, the blood culture result and urine culture result were also consistent with *E. coli*. Meropenem (*E. coli* isolates were susceptible to meropenem) was then administered systemically. Error bars indicate triplicate testing. (**B**) Case 2: An 88-year-old woman was diagnosed with sepsis associated with retrograde cholangitis. The Tm mapping result (Difference Value > 0.5) was polymicrobial infection, and 2 days later, the blood culture result was found to be *Klebsiella oxytoca*, *Haemophilus influenzae*, and *S. pneumoniae*. The blood bacterial concentration was thus calculated in conversion as *E. coli*. Cefepime (*K. oxytoca* and *H. influenzae* isolates were susceptible to cefepime. *S. pneumoniae* isolates were intermediate to cefepime) was then administered systemically. Error bars indicate triplicate testing. (**C**) Case 3: A 94-year-old woman was diagnosed with sepsis associated with urinary tract infection. The Tm mapping result (Difference Value = 0.19) was *E. coli*, and 2 days later, the blood culture result and the urine culture result were also consistent with *E. coli*. Tazobactam/piperacillin (*E. coli* isolates were susceptible to tazobactam/piperacillin) was then administered systemically. Error bars indicate triplicate testing. (**D**) Case 4: An 84-year-old woman was diagnosed with sepsis associated with postoperative wound infection after the spine surgery. The Tm mapping result (Difference Value = 0.28) was *S. dysgalactiae*, and 2 days later, the blood culture result was also consistent with *S. dysgalactiae*. Tazobactam/piperacillin (*S. dysgalactiae* isolates were susceptible to tazobactam/piperacillin) was then administered systemically. Error bars indicate triplicate testing. (**E**) Case 5: An 81-year-old woman was diagnosed with sepsis associated with urinary tract infection. The Tm mapping result (Difference Value = 0.48) was *Enterobacter aerogenes*, and 2 days later, the blood culture result and the urine culture result were also consistent with *E. aerogenes* (2 types of mutant strains). Tazobactam/piperacillin (*E. aerogenes* isolates were susceptible to tazobactam/piperacillin) was then administered systemically. Error bars indicate triplicate testing. (**F**) Healthy Control: A 44-year-old woman who does not have any illnesses including infectious diseases and participated in this study as a volunteer. No bacteria were detected in the blood using the Tm mapping method and blood culture. Error bars indicate triplicate testing.

We subsequently identified and quantified pathogenic bacteria in septic blood samples (and a healthy control sample) and then examined the time-dependent changes in the number of bacteria, BT, WBC, CRP, presepsin, and IL-6 values before and 24 and 72 h after antibiotic treatment (Fig. 3). Regarding the identification, to assess the suitability of the Tm mapping method, we established the interpretative criteria based on the Difference Value^5^ (Supplemental Table S3).

- Case 1 (Fig. 3A): A 76-year-old woman was diagnosed with sepsis associated with urinary tract infection. The Tm mapping result (Difference Value = 0.29) was *E. coli*, and 2 days later, the blood culture result and urine culture result were also consistent with *E. coli*. Before antibiotic treatment, the number of *E. coli* was 1,320/mL. Meropenem (*E. coli* isolates were susceptible to meropenem) was then administered systemically. At 24 h after antibiotic treatment, the number of *E. coli* had decreased to 260/mL, although the other current biomarkers had increased, except for IL-6. At 72 h after antibiotic treatment, bacteria were no longer detected in the blood.
- Case 2 (Fig. 3B): An 88-year-old woman was diagnosed with sepsis associated with retrograde cholangitis. The Tm mapping result (Difference Value > 0.5) was polymicrobial infection, which means the causative bacteria could not be identified, and 2 days later, the blood culture result was found to be *Klebsiella oxytoca*, *Haemophilus influenzae*, and *S. pneumoniae*. The blood bacterial concentration was thus calculated in conversion as *E. coli*. Before antibiotic treatment, the number of bacteria was 175,280/mL. Cefepime (*K. oxytoca* and *H. influenzae* isolates were susceptible to cefepime. *S. pneumoniae* isolates were intermediate to cefepime) was then administered systemically. At 24 h after antibiotic treatment, the number of bacteria had decreased to 31,460/mL, although the WBC, CRP, and presepsin values had increased. At 72 h after antibiotic treatment, the number of bacteria had decreased to 2,060/mL.
- Case 3 (Fig. 3C): A 94-year-old woman was diagnosed with sepsis associated with urinary tract infection. The Tm mapping result (Difference Value = 0.19) was *E. coli*, and 2 days later, the blood culture result and the urine culture result were also consistent with *E. coli*. Before antibiotic treatment, the number of *E. coli* was 3600/mL. Tazobactam/piperacillin (*E. coli* isolates were susceptible to tazobactam/piperacillin) was then administered systemically. At 24 h after antibiotic treatment, the number of *E. coli* had decreased to 2100/mL, although the CRP value had increased. At 72 h after antibiotic treatment, bacteria were no longer detected in the blood, although the BT and presepsin values had increased.
- Case 4 (Fig. 3D): An 84-year-old woman was diagnosed with sepsis associated with postoperative wound infection after the spine surgery. The Tm mapping result (Difference Value = 0.28) was *S. dysgalactiae*, and 2 days later, the blood culture result was also consistent with *S. dysgalactiae*. Before antibiotic treatment, the number of *S. dysgalactiae* was 2,660/mL. Tazobactam/piperacillin (*S. dysgalactiae* isolates were susceptible to tazobactam/piperacillin) was then administered systemically. At 24 h after antibiotic treatment, the number of *S. dysgalactiae* had decreased to 300/mL, although the CRP value had increased. At 72 h after antibiotic treatment, the number of *S. dysgalactiae* had decreased to 20/mL, although the BT had increased.
- Case 5 (Fig. 3E): An 81-year-old woman was diagnosed with sepsis associated with urinary tract infection. The Tm mapping result (Difference Value = 0.48) was *Enterobacter aerogenes*, and 2 days later, the blood culture result and the urine culture result were also consistent with *E. aerogenes* (two strains). Before antibiotic treatment, the number of *E. aerogenes* was 41,090/mL. Tazobactam/piperacillin (*E. aerogenes* isolates were susceptible to tazobactam/piperacillin) was then administered systemically. At 24 h after antibiotic treatment, the number of *E. aerogenes* had decreased to 19,145/mL, although the WBC, CRP, and presepsin values had increased. At 72 h after antibiotic treatment, the number of *E. aerogenes* had decreased to 70/mL, although the CRP and presepsin values had increased.
- Healthy Control (Fig. 3F): A 44-year-old woman who does not have any illnesses including infectious diseases and participated in this study as a volunteer. No bacteria were detected in the blood using the Tm mapping method and blood culture, and both CRP and IL-6 were below the detection sensitivity. Furthermore, BT, WBC, and Presepsin were each within the normal range. There was almost no daily variation in each biomarker.

## Discussion

In the present study, we developed a novel rapid method for identifying and quantifying unknown pathogenic bacteria in a clinical sample within four hours of sample collection (Japanese patent No. 7023465 and international patent application No. PCT/JP2018/023597). As the main technology of this method, the Tm mapping method can quickly identify more than 160 species registered in the database^5^ without a blood culture (Japanese patent No. 4590573 and international patent No. WO2007/097323). In this study, the protocol for the Tm mapping method was modified slightly to perform accurate quantification of bacteria (e.g. up to 30 cycles for 1st PCR so that PCR amplification does not saturate and affect quantification, etc.), but this modification did not affect the identification results (Supplemental Table S4). The most important point of this study is that the novel method can be used to determine the number of bacteria in a clinical sample as a novel biomarker of infectious diseases.

To precisely measure the number of bacteria in a clinical sample, in addition to some technical ingenuities described in the Results section, bacterial DNA contamination-free PCR is essential. To perform bacterial DNA contamination-free PCR, not only the eukaryote-made thermostable DNA polymerase^23^ we developed (Japanese patent No. 5583602 and international patent No. WO2010/082640) but also the contamination-free PCR tubes, distilled water, primers, DNA extraction kit, and appropriate experimental environment are indispensable. We achieved bacterial DNA contamination-free PCR with this method and did not detect any contamination during the DNA extraction and PCR steps.

However, another major problem is bacterial DNA contamination of vacuum blood collection tubes (Supplemental Fig. S1). We evaluated the contamination state of commercially available vacuum blood collection tubes, and bacterial DNA (i.e. dead bacteria) was detected in 8 tubes out of 10. While the number of bacteria differed among tubes, the average number was 65 bacteria/blood collection tube, which would hamper attempts to detect a small number of bacteria in a clinical sample accurately. To address this problem, we developed a bacterial DNA contamination-free vacuum blood collection tube (OP-BF0205-1) in cooperation with Nipro Corporation (Osaka, Japan). As a result, no bacteria were detected in any (10 tubes out of 10) of Nipro′s vacuum blood collection tubes (Supplemental Fig. S1). Of note, this blood collection tube is not commercially available at present, and consultation required for ordering.

Through five cases of sepsis patients, we showed that the number of bacteria is a novel useful biomarker that more sensitively reflects the therapeutic effect than currently available biomarkers (BT, WBC, CRP, presepsin, and IL-6). Since the number of bacteria can change greatly over a short period of time, the time-dependent changes in the number of bacteria would be particularly useful for monitoring therapeutic effects. Since the current biomarkers reflect the host's immune response to microorganisms, they will inevitably cause a time-lag. Indeed, the current biomarkers showed an increasing trend at 24 h after antibiotic administration, even though the antibacterial treatment was effective, and the number of bacteria in the blood was decreasing.

The number of bacteria referred to in the novel method is the total number of bacteria in both the plasma and buffy coat, i.e. the total number of bacteria floating in the blood and bacteria phagocytosed by leukocytes. By including the buffy coat, the detection sensitivity is increased. We experimentally confirmed the quantitative ratio of bacteria floating in septic blood to bacteria phagocytosed by leukocytes as around 1:10 (data not shown). Phagocytosis by leukocytes indicates that the bacteria in the buffy coat is a pathogen. Conversely, to determine contamination, it is sufficient to confirm the absence of bacteria in the buffy coat despite the presence of bacteria in the plasma. Another advantage of including the buffy coat is that the number of bacteria fluctuates greatly in a short period of time, reflecting therapeutic effects. As a biomarker of sepsis, the number of bacteria changes every hour due to competition between the speed at which bacteria proliferate and the speed at which bacteria are phagocytosed and destroyed by leukocytes. If the antibacterial treatment is effective, bacterial proliferation stops, and phagocytosis by leukocytes becomes dominant, reducing the number of bacteria in septic blood.

By using the bacterial universal PCR and the established bacterial DNA contamination-free PCR, we were able to rapidly detect the absence of bacteria (below the limit of detection) in a sample. We already showed that there were no bacteria detected in the blood samples of five healthy controls (Table 1). For example, in the case of fever of unknown origin, rapid diagnosis of the absence of bacteria is useful because treatment is completely different depending on whether it is bacterial or not. Furthermore, using the rapid method of identifying and quantifying pathogenic bacteria, we reported that most (approximately 90%) cases with suspected sepsis in our hospital were aseptic, results that were also clinically useful.

In conclusion, our novel method enables the identification and quantification of bacteria in a clinical sample within four hours of whole-blood collection. Based on the number of bacteria in a blood sample, we were able to estimate the severity of microbial infection accurately. Furthermore, using the time-dependent changes in the number of bacteria, we were able to monitor the therapeutic effect accurately. As a result, the number of bacteria may provide clues to decide the better timing to stop antibiotic treatment. This method for the rapid identification and quantification of bacteria may change our approach to medical care.

## Methods

### Clinical specimens

Whole-blood samples (2 mL each) were randomly collected from patients with suspected sepsis. All procedures were performed under a protocol approved by the Ethics Committee at the University of Toyama, and written informed consent was obtained from all patients. The methods were carried out in accordance with the approved guidelines.

### Isolation of bacterial genomic DNA from whole blood

A total of 2 mL of venous blood was collected in EDTA-2K tubes (BD Biosciences Japan, Tokyo, Japan). The blood samples were then centrifuged at 100×*g* for 5 min to spin down the red blood cells, and the resulting supernatant fraction (500 μL) with buffy coat was used. The supernatant with buffy coat was centrifuged again at 20,000×*g* for 10 min, and 400 μL of the supernatant fraction was carefully removed in order to not disturb the pellets. Next, 1 mL of molecular-grade distilled water (water deionized and sterilized for molecular biology; Nacalai Tesque, Inc., Kyoto, Japan) was added to the pellets, and the mixture was gently turned upside down several times, and subsequently centrifuged at 20,000×*g* for 5 min. Finally, 1 mL of the supernatant fraction was again carefully removed in order to not disturb the pellets. DNA was isolated from the pellets using a DNA extraction kit (QIAamp UCP Pathogen Mini Kit; Qiagen, Germany). To lyse bacterial cell walls thoroughly and obtain consistent high detection sensitivity, we modified the supplier’s protocols (mechanical pre-lysis protocol and spin protocol) as follows: Step 1, Add 100 μL of molecular-grade distilled water (water deionized and sterilized for molecular biology; Nacalai Tesque, Inc.) to a tube containing a bacterial pellet, or as a negative control for DNA extraction, add 200 μL of molecular-grade distilled water to an empty tube; then, mix with a vortex mixer for 10 s, and spin it down briefly in a microcentrifuge. Step 2, Transfer the sample from Step 1 into a fresh Pathogen Lysis Tube (S) containing small beads to assist in lysing bacteria; then, add 400 μL of Buffer ATL (containing Reagent DX), and mix the sample with a vortex mixer for 10 min at maximum speed. Step 3, Add 40 μL of Proteinase K and mix the sample by pipetting up and down several times, and then incubate the sample at 56 °C for 10 min. Step 4, Add 200 μL of Buffer APL2 to the sample, mix with a vortex mixer for 10 min at maximum speed, and then incubate the sample at 70 °C for 10 min. Step 5, Briefly spin the tube (Pathogen Lysis Tube (S)) to remove droplets from the inside of the lid. Step 6, Transfer 740 μL of the supernatant from Step 5 into a fresh 1.5-mL microcentrifuge tube, taking care to not transfer any of the small beads in doing so. Step 7, Add 300 μL of ethanol to the lysate. Close the cap, and mix thoroughly by pulse-vortex mixing for 15–30 s.

We conducted the remaining steps of the procedure in accordance with the supplier’s instructions, but during the final elution step, we added 50 μL of Buffer AVE (elution buffer) incubated at room temperature for 10 min and then centrifuged the sample at full speed for 1 min to elute the DNA. We repeated the elution step again, so the final amount of eluted DNA solution was 100 μL.

### PCR assays

The following is a nested PCR procedure (first PCR: 30 cycles → dilute 100-fold → second, nested PCR: 35 cycles). The Rotor-Gene Q (Qiagen) was used for the amplification, real-time detection of the target DNA, and Tm value analysis of the amplified products. All PCR assays were performed as single-tube assays (no multiplex PCR). We used 1.5-mL PCR-clean Eppendorf tubes that were RNase- and DNase-free (Eppendorf, Germany), 0.2-mL PCR tubes (Qiagen) for the first PCR, and 0.1-mL Strip Tubes and Caps (Qiagen) for the second (nested) PCR.

All oligonucleotide primers for Tm mapping identification and quantification were designed using a multiple alignment software program (ClustalX) and synthesized by Life Technologies Japan, Ltd. (Tokyo, Japan). Bacterial universal primers were designed to universally amplify the seven regions of the bacterial 16S ribosomal RNA gene (16S rDNA). The primers were as follows: Region 1 primers (forward 1a: 5′-AGAGTTTGATCATGGCTCAG-3′, forward 1b: 5′-AGAGTTTGATCCTGGCTCAG-3′, reverse: 5′-CGTAGGAGTCTGGACCGT-3′, amplicon size: 338 bp. For the first PCR, forward 1a and 1b are equally mixed together, but for the second PCR, only forward 1a is used), Region 2 primers (forward: 5′-GACTCCTACGGGAGGCA-3′, reverse: 5′-TATTACCGCGGCTGCTG-3′, amplicon size: 199 bp), Region 3 primers (forward: 5′-AGCAGCCGCGGTAATA-3′, reverse: 5′-GGACTACCAGGGTATCTAATCCT-3′, amplicon size: 287 bp), Region 4 primers (forward: 5′-AACAGGATTAGATACCCTGGTAG-3′, reverse: 5′-AATTAAACCACATGCTCCACC-3′, amplicon size: 181 bp), Region 5 primers (forward: 5′-TGGTTTAATTCGATGCAACGC-3′, reverse: 5′-GAGCTGACGACAGCCAT-3′, amplicon size: 120 bp), Region 6 primers (forward: 5′-TTGGGTTAAGTCCCGC-3′, reverse: 5′-CGTCATCCCCACCTTC-3′, amplicon size: 109 bp), and Region 7 primers (forward: 5′-GGCTACACACGTGCTACAAT-3′, reverse: 5′-CCGGGAACGTATTCACC-3′, amplicon size: 166 bp).

The first PCR primer set was the same as the Region 1 forward primer (forward 1a and 1b are equally mixed together) and the Region 7 reverse primer. The second PCR primer set for quantification was the same as the Region 3 primers.

During the first PCR procedure, the PCR reaction mixture (20 µL) contained 10 µL of DNA template in 200 µM of each dNTP (CleanAmp™ Hot Start dNTP Mix, Sigma-Aldrich, USA) filtered using an Amicon Ultra 50K centrifugal filter (Merck Millipore, Germany), 50 mM KCl, 2.25 mM MgCl_2_, 10 mM Tris–HCl (pH 8.3), 0.3 µM of each primer, 1 × EvaGreen (Biotium Inc., CA, USA), and 1.0 units (0.5 µL) of eukaryote-made thermostable DNA polymerase supplemented with stock buffer solution. The generation of eukaryote-made thermostable DNA polymerase using *Saccharomyces cerevisiae* has been described previously^23^. In place of 10 µL of DNA template, the PCR reaction mixture contained 10 µL of DNA extracted from 3 serially diluted concentrations (2500 cells/10 µL = 50,000 cells/mL of whole blood, 250 cells/10 µL = 5000 cells/mL of whole blood, and 12.5 cells/10 µL = 250 cell/mL of whole blood, counted using flow cytometry method) of *E. coli* (ATCC 25922) as quantification standards in quantitative real-time PCR, or 10 µL of molecular-grade distilled water (water deionized and sterilized for molecular biology; Nacalai Tesque, Inc.) as a negative control for the PCR step.

Each sample was incubated for 5 min at 95 °C to activate the Hot Start dNTPs and then was denatured for 10 s at 94 °C, annealed for 10 s at 57 °C, extended for 30 s at 72 °C and subjected to fluorescence acquisition for 2 s at 82 °C for 30 cycles. The PCR product was diluted 100-fold with molecular-grade distilled water (water deionized and sterilized for molecular biology; Nacalai Tesque, Inc.) and then used as a template for the second (nested) PCR procedure.

For the second (nested) PCR procedure, the PCR reaction mixture (20 µL) contained 10 µL of DNA template of the diluted first PCR product in 200 µM of each dNTP (CleanAmp™ Hot Start dNTP Mix; Sigma-Aldrich) filtered using an Amicon Ultra 50K centrifugal filter (Merck Millipore), 50 mM KCl, 2.5 mM MgCl_2_, 10 mM Tris–HCl (pH 8.3), 0.25 µM of each primer, 1 × EvaGreen (Biotium, Inc.), and 1.0 units (0.5 µL) of eukaryote-made thermostable DNA polymerase supplemented with stock buffer solution. The 7 samples used to amplify Regions 1 to 7 were incubated for 5 min at 95 °C to activate the Hot Start dNTPs and then denatured for 10 s at 94 °C, annealed for 10 s at 57 °C, extended for 10 s at 72 °C, and subjected to fluorescence acquisition for 2 s at 82 °C for 35 cycles. To quantify bacteria in a sample, the threshold cycle (Ct) values amplified by Region 3 primers in the second PCR were analyzed using the Rotor-Gene Q software program. The seven PCR amplicons were then analyzed to obtain the Tm values. If no amplification was observed by the 35th cycle of all 7 secondary PCRs, we defined the sample as containing no bacteria.

### The melting temperature (Tm) value analysis

For the Tm value analysis, the resulting 7 amplicons were heated at 95 °C for 10 s and then cooled at 72 °C for 90 s. A post-PCR Tm value analysis was performed from 72  to 95 °C, increasing at 0.5 °C/step. The data profile was subsequently analyzed using the Rotor-Gene Q software program, and the Tm values were identified. Finally, the dominant bacteria in a sample were identified using the Tm mapping method.

### Quantification of bacterial concentration in a blood sample

We conducted quantitative real-time PCR for the relative quantification of bacteria in blood samples. In every test, the standard curve was formed by Ct values (region 3 amplicon) of three serially diluted quantification standards (*E. coli* DNA) with known concentrations measured by flow cytometry. The blood bacterial concentrations were measured using the standard curve and finally adjusted according to the 16S ribosomal RNA operon copy number of pathogenic bacteria identified by the Tm mapping method.

### Culture-based biochemical identification of bacteria

A total of 20 mL of whole blood samples (for one aerobic blood culture bottle and one anaerobic blood culture bottle, respectively) were collected simultaneously with 2 mL of whole blood sample for Tm mapping method from the same puncture site. The whole-blood samples were then analyzed according to standard methods used by the Clinical Laboratory Center (certified ISO15189) at Toyama University Hospital. The blood culture procedures were performed using the BacT/ALERT 3D system (bioMerieux, Inc., Mercy-l’Etoile, France). Positive blood culture bottles were subcultured onto sheep blood agar, chocolate agar, and BTB lactose-contained agar. Moreover, as a result of microscopic examination, added appropriate media if necessary. Then, they were incubated aerobically or anaerobically until sufficient growth was present to proceed with testing (usually 18–24 h). The specific identification methods differed according to the organism, although they involved the MicroScan WalkAway system (Siemens Healthcare Diagnostics, IL, USA), RapID ANA II (Thermo Fisher Scientific, UK), and various latex agglutination and biochemical spot tests.

### Supplementary Information

Supplementary Information.

## Data Availability

All data generated or analyzed during this study are included in this published article and its supplementary information files.
