# Supplementary material for: Novel rapid method for identifying and quantifying pathogenic bacteria within four hours of blood collection
Source: Sci Rep. 2024 Jan 12;14:1199. doi: 10.1038/s41598-023-50864-0 (PMC10786899; doi:10.1038/s41598-023-50864-0)
Supplement: Supplementary file 1 — Supplementary Information. [file 41598_2023_50864_MOESM1_ESM.docx]

***Supplementary Information***

**Novel Rapid Method for Identifying and Quantifying Pathogenic Bacteria within Four Hours of Blood Collection**

Akio Miyakoshi^1^†, Hideki Niimi^2^*†, Tomohiro Ueno^2^, Masahiro Wakasugi^3^, Yoshitsugu Higashi^4^, Yuki Miyajima^4^, Masashi Mori^5^, Homare Tabata^6^, Hiroshi Minami^6^, Akinori Takaoka^7^, Atsushi Hayashi^1^, Yoshihiro Yamamoto^4^, and Isao Kitajima^8^

1. Department of Ophthalmology, Toyama University Hospital

2. Clinical Laboratory Center, Toyama University Hospital

3. Disaster and Emergency Center, Toyama University Hospital

4. Department of Clinical Infectious Diseases, Toyama University Hospital

5. Research Institute for Bioresources and Biotechnology, Ishikawa Prefectural University

6. Life Science Center, Hokkaido Mitsui Chemicals, Inc.

7. Institute for Genetic Medicine, Hokkaido University

8. Administrative office, University of Toyama

*Correspondence to:

Hideki Niimi, M.D. Ph.D. E-mail address: hiniimi@med.u-toyama.ac.jp

Clinical Laboratory Center, Toyama University Hospital,

2630 Sugitani Toyama 930-0194 JAPAN

Phone: (+81)-76-434-7759, Fax: (+81)-76-434-7759

† Both authors contributed equally to this work.

**Supplemental Table S1. Variations in the 16S ribosomal RNA operon copy number in genomes**

| Name of *Mycoplasma*, *Ureaplasma* and other bacteria | 16S ribosomal RNA  operon copy number |
| --- | --- |
| *Bacillus cereus* | 13 |
| *Clostridium difficile* | 12 |
| *Aeromonas hydrophila* | 10 |
| *Clostridium perfringens* | 10 |
| *Enterobacter aerogenes* | 8 |
| *Enterobacter cloacae* | 8 |
| *Klebsiella pneumoniae* | 8 |
| *Bacteroides vulgatus* | 7 |
| *Escherichia coli* | 7 |
| *Streptococcus agalactiae* | 7 |
| *Bacteroides fragilis* | 6 |
| *Enterococcus faecium* | 6 |
| *Bacteroides distasonis* | 5 |
| *Staphylococcus aureus* | 5 |
| *Staphylococcus epidermidis* | 5 |
| *Staphylococcus haemolyticus* | 5 |
| *Staphylococcus lugdunensis* | 5 |
| *Enterococcus faecalis* | 4 |
| *Lactobacillus acidophilus* | 4 |
| *Lactobacillus crispatus* | 4 |
| *Paptostreptococcus magnus* | 4 |
| *Paptostreptococcus prevotii* | 4 |
| *Pseudomonas aeruginosa* | 4 |
| *Streptococcus mitis* | 4 |
| *Eubacterium lentum* | 3 |
| *Campylobacter jejuni* | 3 |
| *Propionibacterium acnes* | 3 |
| *Acinetobacter calcoaceticus* | 2 |
| *Gardnerella vaginalis* | 2 |
| *Ureaplasma parvum* | 2 |
| *Ureaplasma urealyticum* | 2 |
| *Mycoplasma hominis* | 2 |
| *Mycoplasma genitalium* | 1 |
| *Mycoplasma pneumoniae* | 1 |

(Copyright: Ueno T. et al. PLoS One. 2015 Jun 4;10(6):e0129032.)

**Supplemental Table S2. Validation of the measurement errors among 10 different quantification trials using the same *Escherichia coli* (ATCC25922) solution**

| Trial No. | Bacterial count  /PCR tube | Difference from the average | Measurement error  from the average (%) |
| --- | --- | --- | --- |
| 1 | 304 | -14.1 | -4.43 |
| 2 | 311 | -7.1 | -2.39 |
| 3 | 326 | 7.9 | 2.48 |
| 4 | 325 | 6.9 | 2.17 |
| 5 | 303 | -15.1 | -4.75 |
| 6 | 311 | -7.1 | -2.23 |
| 7 | 322 | 3.9 | 1.23 |
| 8 | 322 | 3.9 | 1.23 |
| 9 | 333 | 14.9 | 4.68 |
| 10 | 324 | 5.9 | 1.85 |
| average | 318.1 |  |  |
| range | 303 to 333 | -15.1 to 14.9 | -4.75 to 4.68 |

**Supplemental Table S3. Interpretative criteria for the Tm mapping method**

| **Difference Value (D)** | **Suitability for identification** | **Identification** | **Interpretation** |
| --- | --- | --- | --- |
| 0.0 ≤ D ≤ 0.28 | High | All identification results within this range have the same possibility of being the bacterial isolate | Matched the bacteria registered in the database |
| 0.28 < D ≤ 0.5 | Medium  to  Low | The identification result with the lowest Difference Value is highly likely to be the bacterial isolate | Mutant strain, or  Polymicrobial infection*^1^ |
| 0.5 < D | Not suitable | Does NOT identify the bacterial isolate | Polymicrobial infection*^2^, or  Not registered in the database, or Under the limit of identification*^3^ |

(Copyright: Niimi H. et al. Scientific Reports, 2015 Jul 28; 5:12543)

*1: Polymicrobial infection with one dominant bacterial species

*2: Polymicrobial infection with no dominant bacterial species

*3: Under the limit of identification but over or equal to the limit of detection

**Supplemental Table S4. A comparison of the identification results between the original protocol for the Tm mapping method and the modified protocol for the Tm mapping quantification method**

|  | Using the original protocol  for the Tm mapping method | | | | Using the modified protocol  for the Tm mapping quantification method | | | |
| --- | --- | --- | --- | --- | --- | --- | --- | --- |
| DNA template | 2 μL | | | | 10 μL | | | |
| 1^st^ PCR | 40 cycles | | | | 30 cycles | | | |
| Dilution | 1/ 500 | | | | 1/ 100 | | | |
| 2^nd^ PCR | 30 cycles | | | | 35 cycles | | | |
| Trials | Tm values of 1^st^ trial | Tm values of 2^nd^ trial | Tm values of 3^rd^ trial | Tm values of NC | Tm values of 1^st^ trial | Tm values of 2^nd^ trial | Tm values of 3^rd^ trial | Tm values of NC |
| Region 1 | 89.50 | 89.60 | 89.50 | N.D | 89.60 | 89.60 | 89.75 | N.D. |
| Region 2 | 88.85 | 88.85 | 88.60 | N.D. | 88.85 | 88.85 | 88.90 | N.D. |
| Region 3 | 89.75 | 90.00 | 89.65 | N.D. | 89.90 | 89.90 | 89.85 | N.D. |
| Region 4 | 90.25 | 90.25 | 90.15 | N.D. | 90.25 | 90.25 | 90.25 | N.D. |
| Region 5 | 86.75 | 86.75 | 86.40 | N.D. | 86.75 | 86.75 | 86.65 | N.D. |
| Region 6 | 88.10 | 88.15 | 87.85 | N.D. | 88.10 | 88.10 | 88.15 | N.D. |
| Region 7 | 87.75 | 87.90 | 87.90 | N.D. | 88.00 | 87.90 | 88.25 | N.D. |
| Identification results | *E. coli* | *E. coli* | *E. coli* | N.D. | *E. coli* | *E. coli* | *E. coli* | N.D. |
| Diff. Value | 0.24 | 0.26 | 0.19 | - | 0.25 | 0.19 | 0.24 | - |

We checked the difference between the identification results using the original protocol for the Tm mapping method and the modified protocol for the Tm mapping quantification method. As a result, no significant differences were observed between the original and modified protocols.

**NC** = negative control, **Diff. Value** = Difference Value, **N.D.** = not detected

**Supplemental Figure S1. Bacterial contamination in vacuum blood collection tubes**

**
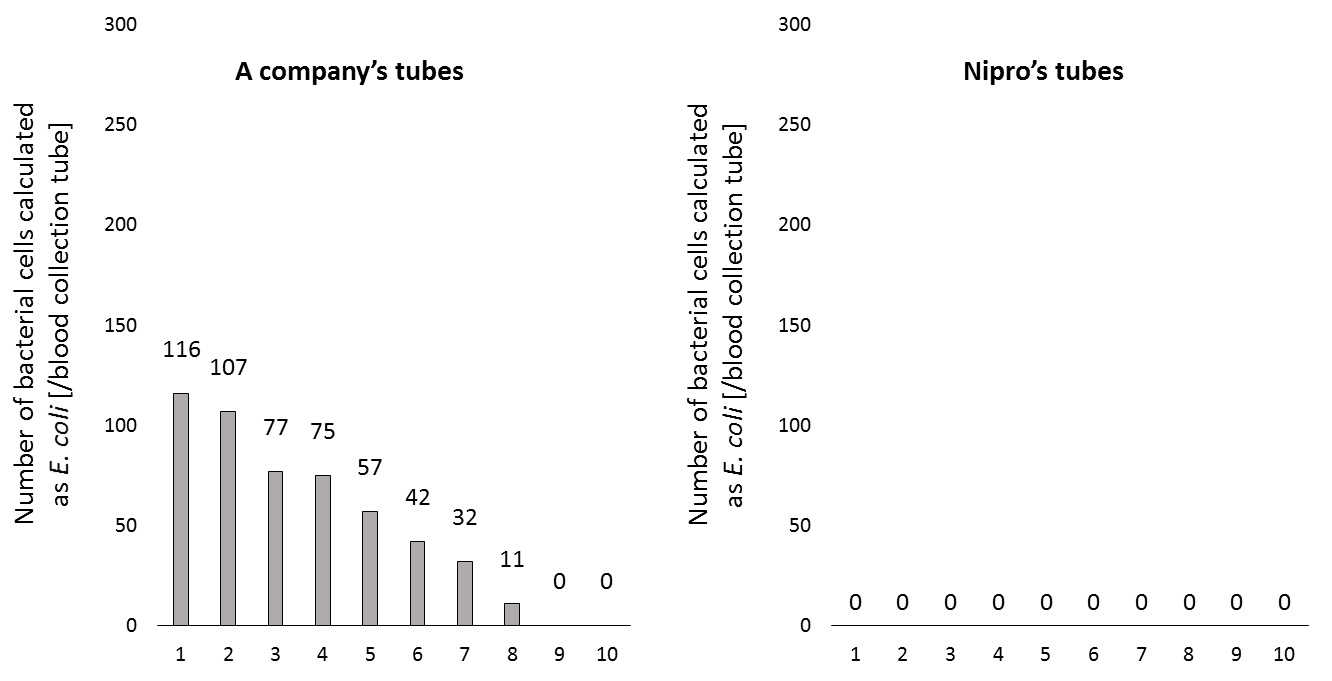
**

Vacuum blood collection tubes (n = 10)

Vacuum blood collection tubes (n = 10)

To check whether or not vacuum blood collection tubes were contaminated with bacterial DNA, we added 2 mL each of molecular-grade distilled water to 10 vacuum blood collection tubes (EDTA-2K) and tried to detect and quantify the bacterial DNA in the tubes. We performed pelletizing, DNA extraction, and a quantitative polymerase chain reaction assay according to the protocol described in the Methods section. As a result, bacterial DNA were detected in 8 of the 10 commercially available tubes. Those bacteria could not be identified using the Tm mapping method (Diff. Value > 0.5) as they were not registered in the database, so the bacterial concentrations were quantified as *Escherichia coli*. The average number of bacteria was 65/blood collection tube. In contrast, no bacteria were detected in any of Nipro′s tubes (OP-BF0205-1; bacterial DNA contamination-free vacuum blood collection tubes). Error bars indicate triplicate testing.
